# Dietary antioxidant intake in school age and lung function development up to adolescence

**DOI:** 10.1183/13993003.00990-2019

**Published:** 2020-02-20

**Authors:** Emmanouela Sdona, Jenny Hallberg, Niklas Andersson, Sandra Ekström, Susanne Rautiainen, Niclas Håkansson, Alicja Wolk, Inger Kull, Erik Melén, Anna Bergström

**Affiliations:** 1 Institute of Environmental Medicine, Karolinska Institutet, Stockholm, Sweden; 2 Sachs’ Children's Hospital, Södersjukhuset, Stockholm, Sweden; 3 Dept of Clinical Science and Education, Södersjukhuset, Karolinska Institutet, Stockholm, Sweden; 4 Centre for Occupational and Environmental Medicine, Region Stockholm, Stockholm, Sweden; 5 Global and Sexual Health, Dept of Public Health Sciences, Karolinska Institutet, Stockholm, Sweden; 6 Division of Preventive Medicine, Brigham and Women's Hospital, Boston, MA, USA; 7 Dept of Surgical Sciences, Uppsala University, Uppsala, Sweden

## Abstract

Dietary antioxidant intake has been hypothesised to influence lung function. The association between total antioxidant capacity (TAC) of the diet at age 8 years and lung function development up to 16 years in 2307 participants from the Swedish population-based birth cohort BAMSE (Children, Allergy, Milieu, Stockholm, Epidemiology) was investigated.

Information on TAC was obtained from a food frequency questionnaire at 8 years. Lung function was measured by spirometry at 8 and 16 years, impulse oscillometry (IOS) and exhaled nitric oxide fraction (*F*_eNO_) at 16 years. Low lung function was defined as forced expiratory volume in 1 s (FEV_1_) z-score below the 25th percentile. Longitudinal associations between TAC and lung function were analysed by mixed effect models adjusted for potential confounders. Stratification by asthma at 8 years was performed to examine effect modification.

The median TAC intake was 10 067 μmol Trolox equivalents (TE)·g^−1^, with males having a lower mean compared to females (9963 *versus* 10 819 μmol TE·g^−1^). In analyses of lung function change between 8 and 16 years, there were no statistically significant associations between TAC in tertiles and spirometry results for the total study population. Among children with asthma at 8 years (prevalence 7%), higher TAC was associated with higher mean FEV_1_ (0.46 sd, 95% CI 0.11–0.80) and decreased odds of low lung function at 16 years (OR 0.28, 95% CI 0.12–0.65). There were no associations between TAC and forced vital capacity or IOS/*F*_eNO_ results.

High dietary antioxidant intake in school age may be associated with improved lung function development from school age to adolescence among children with asthma.

## Abstract 2

**Dietary antioxidant intake at school age may influence lung function development as measured by FEV_1_ up to adolescence among children with asthma. In contrast, no association was observed among children without asthma.** http://bit.ly/2CzEZ8W

## Introduction

In recent years, the importance of a full growth to maximal lung function in childhood has been reinforced by accumulating evidence that lung function deficits established by school age may track into adult life [1, 2]. Thus, achieving optimal lung function is an important goal in the prevention of chronic respiratory diseases and subsequent mortality, and a major public health objective [3]. However, less is known about factors that might influence lung function trajectories [4, 5].

The association between dietary factors with antioxidant and anti-inflammatory properties and risk of asthma and other chronic respiratory diseases in the general population has been investigated previously [6–8]. Prospective studies examining the association between maternal diet during pregnancy and the occurrence of asthma and other allergic diseases in the offspring have contributed information on the role of dietary exposures early in life [9]. Analyses from the Swedish BAMSE birth cohort also show that a high intake of dietary antioxidants at age 8 years was associated with a reduced risk of subsequent development of IgE sensitisation to inhalant allergens and allergic asthma [10]. A recent prospective study from Japan found a significant inverse association between fruit intake and the onset of respiratory allergic symptoms in schoolchildren [11].

Epidemiological studies on the association between dietary antioxidants and lung function show conflicting results [12–14]. Most studies have been cross-sectional, but a prospective study in middle-aged adults from three participating countries of the European Community Respiratory Health Survey indicated that a higher intake of fruits and tomatoes was associated with a slower decline in lung function 10 years later [15]. A case–control study in Puerto Rican children indicated that a diet with frequent consumption of vegetables and grains and low consumption of dairy products and sweets was associated with higher lung function, as measured by forced expiratory volume in 1 s (FEV_1_) and forced vital capacity (FVC) [16].

Longitudinal studies on childhood diet and subsequent lung function development are still lacking. Thus, it remains unclear if diet at school age influences lung function. The aim of this study was to investigate the association between dietary antioxidant intake at age 8 years and lung function development between 8 and 16 years. In order to estimate the cumulative action of the antioxidants present in foods, total antioxidant capacity (TAC) of the diet was used [10].

## Methods

### Study population and study design

The study was conducted within the population-based birth cohort BAMSE (Swedish abbreviation for Children, Allergy, Milieu, Stockholm, Epidemiology), in which 4089 children (born 1994–1996) from predefined areas of Stockholm County, Sweden have been followed repeatedly from infancy [4, 17]. In brief, baseline information was collected through parental questionnaires when the children were aged 2 months on average and follow-up questionnaires eliciting information on symptoms of allergic diseases and selected exposures were answered by the parents when the children were aged 1, 2, 4 and 8 years and by the adolescents themselves at 16 years. At ages 8 and 16 years, participants were invited to clinical examinations, which included anthropometric measurements, lung function testing and blood sampling using standardised methods. Sera were analysed for specific IgE to common inhalant and food allergens. Drop-out rates remained low at all ages, and at 16 years 78% (n=3180) completed the questionnaire and 62% (n=2547) attended the clinical examination. The BAMSE study and respective follow-ups were approved by the regional ethical review board (Karolinska Institutet, Stockholm, Sweden), and written informed consent was obtained from parents at 8 years and study participants at 16 years.

### Dietary assessment

Diet was assessed at 8 years, using a food frequency questionnaire (FFQ). The FFQ was most often filled out by a parent (57%) or by a parent together with the child (40%) and included questions about 98 foods and beverages commonly consumed in Sweden. Children (n=2614) were asked how often, on average, they had consumed each type of food or beverage during the past 12 months. There were 10 prespecified response categories ranging from “never” to “≥3 times per day”. Calculation of the TAC of the FFQ items has been described previously [10]. Briefly, individual TAC estimates were obtained by combining the information on frequency of consumption of specific food items with information from a database of common foods analysed with the oxygen radical absorbance capacity (ORAC) method [18] on the average ORAC content (μmol Trolox equivalents (TE) per day) of age-specific portion sizes. ORAC values were further energy-adjusted using the residuals method [19]. There were 35 food items (including 20 fruits and vegetables) with available ORAC values, while there was no available information on TAC from dietary supplements.

### Lung function testing

Details of lung function testing have been described elsewhere [4]. Briefly, lung function was measured by spirometry at 8 years (n=1832) using a 2200 Pulmonary Function Laboratory (SensorMedics, Anaheim, CA, USA) and by impulse oscillometry (IOS) (n=2452) followed by spirometry at 16 years (n=2056) using a Jaeger MasterScreen-IOS system (Carefusion Technologies, San Diego, CA, USA). The same spirometry test protocol was used at both time points. All participants performed repeated maximal expiratory flow volume (MEFV) measurements. The highest values of FEV_1_ and FVC were extracted and used for analysis, provided that the subject's effort was accepted as being maximal by the test leader, the MEFV curve passed visual quality inspection and the two highest FEV_1_ and FVC readings were reproducible according to American Thoracic Society/European Respiratory Society criteria [20]. FEV_1_/FVC ratios were calculated and expressed as percentages. Standard deviation scores (z-scores) for FEV_1_, FVC and FEV_1_/FVC ratio were computed accounting for age, sex, height and ethnicity [21]. Regarding IOS measurements, the mean value of resistance at 5 and 20 Hz, frequency dependence of resistance and the square root of the area of reactance were used for analyses. Measurements of exhaled nitric oxide fraction (*F*_eNO_) were performed at 16 years (n=2087) at an expiratory flow of 50 mL·s^−1^, using an online chemiluminescent analyser (CLD88; Eco Medics AG, Duernten, Switzerland). Details of lung function measurements, as well as asthma and other definitions are described in the supplementary material.

### Statistical analyses

Differences between children who were included and excluded from the study population were analysed by Chi-squared and t-test, for categorical and continuous variables, respectively. The distribution of selected exposure characteristics by tertiles (T1, T2, T3) of TAC (linear relationship not assumed) was compared using Chi-squared test (categorical covariates) and ANOVA (continuous covariates). Multivariate linear regression on the mean was used to analyse associations between dietary TAC in tertiles at age 8 years and lung function parameters at ages 8 and 16 years. Tests for trends were performed by assigning the median value of dietary TAC within each tertile and tested as a continuous variable in the model. Analyses were stratified by sex and potential interactions with sex were tested by the Wald test using an interaction term between TAC and sex in the statistical model. Covariates were identified from previous literature [22] and included maternal age <26 years (yes or no), older siblings (one or more older sibling at birth, yes or no), socioeconomic status (categorised on the basis of parents' occupation as manual and non-manual workers), parental allergic disease (any maternal or paternal history of asthma or hay fever, yes or no), maternal smoking during pregnancy (yes or no) and parental smoking in infancy (yes or no). Additional adjustment for educational level, energy intake, dietary vitamin D and fish intake, supplement use, obesity, physical activity and active smoking at 16 years did not influence the results and was not included in the final models. In order to assess effect modification, stratified analysis by asthma status at 8 years was conducted based on *a priori* determination. In stratified analysis, the two top tertiles were combined due to small numbers. Sensitivity analysis was conducted using other asthma definitions and symptoms, adjusting for inhaled steroid use, as well as excluding supplement users and children who reported avoidance of fruits or vegetables due to allergic symptoms.

Associations between TAC in tertiles at 8 years and spirometry results (main outcome) up to 16 years were further analysed longitudinally by mixed-effects linear regression with a random intercept, an unstructured correlation matrix and restricted maximum likelihood estimation. An interaction term between TAC and the time indicator variable was incorporated into the model to estimate age-specific associations at 8 and 16 years and changes in lung function between 8 and 16 years. For low lung function (binary variable), defined as FEV_1_ z-score below the 25th percentile (Q1) due to small numbers, logistic regression analysis was used. IOS and *F*_eNO_ results were analysed on the median using quantile regression, due to non-normally distributed data.

Participants who answered the questionnaire with baseline information and follow-up questionnaires at 8 and 16 years, and had a FFQ with a mean energy intake within ±3 log sd, as well as anthropometric and lung function measurements at 8 and/or 16 years were included in the present study. In total, 2307 participants fulfilled these criteria (supplementary figure S1).

All analyses were performed using the statistical software Stata (version 13; StataCorp, College Station, TX, USA).

## Results

### Descriptive results on exposure and outcomes

The children included in the study population (n=2307) were comparable to the excluded children (n=1782) with regard to distribution of selected characteristics (supplementary table S1). At 8 years, the median TAC intake was 10 067 μmol TE·g^−1^, which corresponds approximately to two servings of apples per day [10], with males having an 8% lower mean TAC intake compared to females (9963 *versus* 10 819 μmol TE·g^−1^, p<0.001). Children with older siblings and children who came from a household with university education level at baseline had a significantly higher TAC intake than those without older siblings and from a household with lower education. Additionally, children with higher TAC intake tended to use less inhaled steroids (table 1).

**TABLE 1:** Distribution of selected characteristics in the study population in relation to the total antioxidant capacity (TAC) of the diet (n=2307)

	**Tertiles of the TAC of the diet**^#^			**p-value**^¶^
	**T1**	**T2**	**T3**	
**Subjects**	750	779	778	
**ORAC μmol TE per day**	6946 (1768–8615)	10009 (8615–11477)	13530 (11477–33097)	
**Categorical variables**				
Male	433 (57.7)	375 (48.1)	343 (44.1)	<0.001
Maternal age <26 years	62 (8.3)	52 (6.7)	45 (5.8)	0.153
Parental allergic disease	251 (33.7)	252 (32.5)	223 (29.1)	0.142
High socioeconomic status	631 (85.4)	658 (85.1)	667 (86.7)	0.625
University education	393 (52.4)	423 (54.4)	457 (58.7)	0.038
Maternal smoking during pregnancy	94 (12.5)	87 (11.2)	90 (11.6)	0.701
Parental smoking during infancy	161 (21.5)	150 (19.4)	160 (20.7)	0.572
Older siblings	316 (42.1)	373 (47.9)	391 (50.3)	0.005
Age 8 years				
Overweight and obesity	129 (17.2)	157 (20.2)	170 (21.9)	0.070
Physical activity >2 times per week	119 (15.9)	113 (14.5)	112 (14.4)	0.646
Asthma^+^	65 (8.8)	54 (7.0)	44 (5.7)	0.064
Inhaled steroid use in the past 12 months	74 (9.9)	58 (7.5)	51 (6.6)	0.046
Inhalant IgE sensitisation	186 (26.3)	210 (28.7)	170 (23.2)	0.058
Food IgE sensitisation	146 (20.6)	156 (21.3)	137 (18.7)	0.455
Allergy to fruits and vegetables^§^	88 (12.8)	67 (9.3)	66 (9.3)	0.046
Use of multivitamins	315 (42.5)	316 (41.1)	334 (43.6)	0.622
Fish intake ≥2 times per week	269 (36.0)	293 (37.7)	321 (41.5)	0.079
Age 16 years				
Overweight and obesity	128 (19.1)	103 (14.5)	111 (15.6)	0.056
Asthma	71 (10.1)	51 (6.9)	62 (8.4)	0.091
Inhaled steroid use in the past 12 months	82 (11.3)	53 (7.1)	52 (6.8)	0.003
Inhalant IgE sensitisation	314 (47.9)	311 (44.7)	285 (40.7)	0.029
Food IgE sensitisation	108 (16.5)	85 (12.2)	87 (12.4)	0.039
Active smoking	76 (10.4)	86 (11.4)	92 (12.1)	0.597
**Continuous variables**				
Energy intake kcal	1911.7±467.0	1915.5±451.5	1889.1±464.7	0.474

Data are presented as n, median (range), n (%) or mean±sd, unless otherwise stated. ORAC: oxygen radical absorbance capacity; TE: Trolox equivalents. ^#^: TAC intake (μmol TE per day) as measured with ORAC assay, energy-adjusted to 1900 kcal per day, presented in tertiles (T1, T2, T3); ^¶^: p-values were calculated from the Chi-squared test for categorical variables and ANOVA for continuous variables; ^+^: defined based on the parental questionnaire at age 8 years as more than three episodes of wheeze in the past 12 months and/or at least one episode of wheeze in the past 12 months, in combination with inhaled steroids occasionally or regularly; ^§^: allergic symptoms related to fruits and/or vegetables or avoidance of any fruit or vegetable due to allergic symptoms.

Distribution of anthropometric and lung function characteristics among children in the 8- and 16-year examination is shown in supplementary tables S2 and S3.

### Associations between dietary TAC at 8 years and lung function at 8 and 16 years

In linear regression analyses, associations between dietary TAC in tertiles at 8 years and spirometry results at 8 and 16 years were not statistically significant, although higher mean FEV_1_ and FVC were observed for males (supplementary table S4). Tests for trend or interaction with sex were not statistically significant.

**FIGURE 1:**
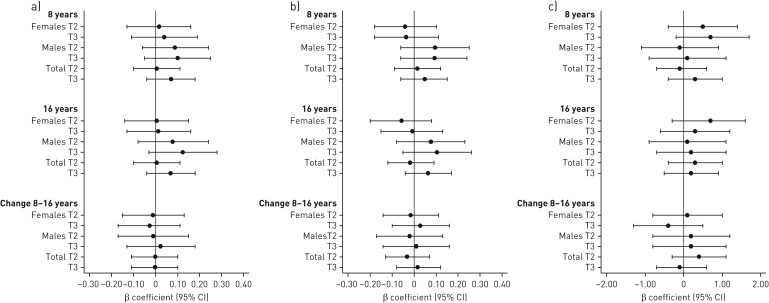
Associations between total antioxidant capacity (TAC) in tertiles (T1 reference, T2, T3) at 8 years and adjusted spirometry results at 8 and 16 years: a) forced expiratory volume in 1 s (FEV_1_) z-score; b) forced vital capacity (FVC) z-score; c) FEV_1_/FVC (%). β-coefficients and 95% confidence intervals were estimated using mixed effect models (n=2115 subjects with 3306 observations), adjusted for maternal smoking during pregnancy, parental smoking during infancy, parental allergic disease, socioeconomic status, older siblings and maternal age <26 years. Totals additionally adjusted for sex.

Figure 1 presents the results from the mixed-effect model analyses of the longitudinal association between dietary TAC at 8 years and lung function up to 16 years. In analyses of lung function change between 8 and 16 years, there were no associations of TAC and spirometry results for the total study population. Consistent with the linear regression results, higher mean FEV_1_ and FVC were observed for males, but associations were not significant.

### Associations between dietary TAC at 8 years and lung function at 16 years by asthma status

To assess possible effect modification, we stratified our analysis by asthma at 8 years. Asthma prevalence in the study population was 7% (n=163) at 8 years; 106 (65%) out of 163 children with asthma also had IgE sensitisation to inhalant and/or food allergens; 134 (82%) had used inhaled steroids occasionally or regularly and 105 (64%) had used bronchodilators in the past 12 months.

Children with asthma had 7% lower mean dietary TAC compared to children without asthma (9708 *versus* 10 450 μmol TE·g^−1^, p<0.01). Among children with asthma at 8 years, higher TAC intake (second and third tertiles combined) at 8 years was associated with higher mean FEV_1_ at 16 years (200.0 mL, 95% CI 38.3–361.6 mL *versus* −7.3 mL, 95% CI −57.2–42.6 mL among children without asthma, p-value for interaction 0.018) (supplementary table S5). This association remained comparable among children with asthma and IgE sensitisation, as well as after adjustment for inhaled steroid use and exclusion of children who reported avoidance of fruits or vegetables due to allergic symptoms (data not shown), and supplement users (supplementary table S6).

**FIGURE 2:**
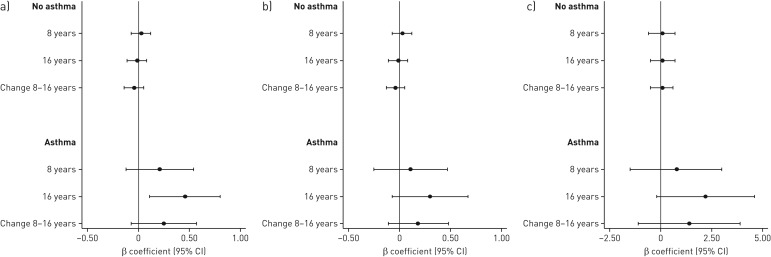
Associations between total antioxidant capacity (TAC) (tertiles 2 and 3 combined *versus* reference tertile 1) at 8 years and adjusted spirometry results at 8 and 16 years stratified by asthma at 8 years: a) forced expiratory volume in 1 s (FEV_1_) z-score; b) forced vital capacity (FVC) z-score; c) FEV_1_/FVC (%). β-coefficients and 95% confidence intervals were estimated using mixed effect models (n=1948 subjects without asthma with 3027 observations and n=154 subjects with asthma with 258 observations), adjusted for sex, maternal smoking during pregnancy, parental smoking during infancy, parental allergic disease, socioeconomic status, older siblings and maternal age <26 years.

In the longitudinal model, higher TAC intake at 8 years was associated with increased FEV_1_ at 16 years (0.46 sd, 95% CI 0.11–0.80) among children with asthma (figure 2). Regarding change in lung function between 8 and 16 years, there is some evidence for increased mean FEV_1_ among children with asthma and higher TAC intake, but results were not statistically significant. In sensitivity analysis using other asthma definitions and symptoms, results were consistent (supplementary table S7). There were no associations among children without asthma, or between TAC intake and FVC.

Low lung function at 16 years (defined as Q1 FEV_1_ z-score) was observed in 36% (46 out of 128) of children with asthma and in 24% (369 out of 1534) of children without asthma. In multivariate logistic regression analysis, higher TAC intake at 8 years was associated with lower odds of low lung function at 16 years among children with asthma (OR 0.28, 95% CI 0.12–0.65), while no association was observed among children without asthma (OR 0.96, 95% CI 0.74–1.25, p-value for interaction between TAC and asthma 0.008) (table 2).

**TABLE 2:** Association between total antioxidant capacity (tertiles (T) 2 and 3 combined *versus* reference T1) at 8 years and lowest quartile (Q1) of forced expiratory volume in 1 s (FEV_1_) at 16 years stratified by asthma at 8 years (n=415)

	**No asthma at 8 years**		**Asthma at 8 years**		**p-value for interaction with asthma**
	**Subjects n**	**OR (95% CI)**	**Subjects n**	**OR (95% CI)**	
**Q1: FEV_1_ z-score**					
T1	118	Reference	24	Reference	0.008
T2 and T3	251	0.96 (0.74–1.25)	22	0.28 (0.12–0.65)	

Logistic regression adjusted for sex, height and age at examination, maternal smoking during pregnancy, parental smoking during infancy, parental allergic disease, socioeconomic status, older siblings and maternal age <26 years.

Finally, higher dietary TAC at 8 years was not associated with any of the measured indices in analyses of lung function using IOS or *F*_eNO_ (supplementary table S8 and table 3).

**TABLE 3:** Associations between total antioxidant capacity (tertiles 2 and 3 combined *versus* reference tertile 1) at 8 years and impulse oscillometry (IOS) and exhaled nitric oxide fraction (*F*_eNO_) results at 16 years stratified by asthma at 8 years

	**No asthma at 8 years**		**Asthma at 8 years**	
	**Subjects n**	**β (95% CI)**	**Subjects n**	**β (95% CI)**
**IOS results**				
*R*_5_ Pa·L^−1^·s	1800	−5.1 (−13.8–3.6)	137	4.9 (−31.0–40.9)
*R*_20_ Pa·L^−1^·s	1800	−0.2 (−7.7–7.2)	137	−9.5 (−37.7–18.6)
*R*_5–20_ Pa·L^−1^·s	1800	−1.5 (−6.3–3.4)	137	−0.7 (−21.9–20.5)
AX^0.5^ (Pa·L^−1^)^0.5^	1799	0.2 (−0.3–0.7)	137	0.5 (−1.7–2.7)
**Additional parameters**				
*F*_eNO_ ppb	1512	0.6 (−0.4–1.7)	117	−4.6 (−15.5–6.4)

IOS and *F*_eNO_ data were analysed by linear regression on the median, adjusted for sex, height and age at examination, maternal smoking during pregnancy, parental smoking during infancy, parental allergic disease, socioeconomic status, older siblings and maternal age <26 years. *R*_5_: resistance at 5 Hz: *R*_20_: resistance at 20 Hz; *R*_5–20_: frequency dependence of resistance; AX^0.5^: square root of the area of reactance.

## Discussion

In our study of 2307 children from a population-based birth cohort, higher TAC intake at 8 years was associated with increased FEV_1_ and decreased odds of low lung function at 16 years among children with asthma. We observed no statistically significant associations between TAC and lung function among children without asthma, or between TAC and other than spirometry measurements.

To our knowledge, this is the first prospective study investigating the association between dietary antioxidant intake in early school age and lung function development from school age to adolescence. Fresh fruits and vegetables are dietary sources rich in antioxidants, such as vitamins and minerals, β-carotene, flavonoids, isoflavonoids and polyphenolic compounds [23]. Respiratory airways are highly susceptible to oxidative damage and antioxidants may protect the airways against oxidants from both endogenous (activated inflammatory cells) and exogenous (indoor and outdoor air pollution, smoke exposure) sources [8]. Previous cross-sectional studies in adults [12–14, 24–26] and children [16, 27], and prospective studies in adults [13, 28, 29] have indicated that higher intake of dietary antioxidants may be associated with better lung function. However, responses to antioxidants might be modified by life stage, genetic susceptibility and environmental sources of oxidative stress [7].

In our study, asthma was an effect modifier in the association between TAC and lung function. Oxidative stress plays a major role in the pathophysiology of asthma, due to chronic activation of airway inflammatory cells and a high intake of antioxidants has been reported to be protective against asthma risk and severity [23, 30]. Moreover, changes in gut microbiome modulated by dietary intake have recently been linked to alterations in immune responses and lung disease [31]. Our results are consistent with a previous prospective study showing that fruit and vegetable intake had a beneficial effect on inflammatory response and lung function in asthmatic children [32]. Moreover, a lower mean TAC intake was observed in children with asthma in our study. This is in line with previous studies showing that children with asthma have lower levels of antioxidants in the serum [32, 33]. Thus, additional antioxidants may have greater impact on children with asthma since they have higher demands. In a recent study on diet and allergic symptoms in children, the protective effect observed from higher intake of fruits and vegetables in children aged 6–7 years was less or not observed in children aged 13–14 years [34].

In our study, we did not observe significant sex differences in the association between TAC and lung function, although males had a lower mean TAC intake compared to females. Sex differences have previously been described among adults, and it was suggested that oxidative stress may be associated with airflow limitation in males, but not in females, due to lower serum antioxidant levels and mediation *via* hormonal mechanisms [35, 36].

A major strength of our study is the population-based longitudinal design and the large sample size with limited loss to follow-up. In contrast to most previous studies that have focused on fruits, vegetables and individual antioxidants [11, 25, 27–29], we used TAC, which reflects the sum of dietary antioxidant intake and takes synergistic and antagonistic effects between compounds into account [10]. Nevertheless, associations with specific nutrients may be diluted using the TAC approach. Additionally, TAC was available only at 8 years and potential dietary changes from 8 to 16 years were not taken into account. Of the 98 food items in the FFQ, 35 had available TAC values, including the most important dietary antioxidant sources, such as fruits, vegetables, whole grains, nuts and chocolate [18]. In contrast, dietary supplements were not included in the calculation of TAC. However, we were able to adjust for use of dietary supplements and several other confounding factors. Moreover, we excluded children who used supplements to control for potential misclassification of exposure and children who reported avoidance of fruits or vegetables due to allergic symptoms to control for potential reverse causality [37], but these exclusions did not affect the observed associations. Lung function was measured using standardised protocols at 8 and 16 years, although lung function measurements post-bronchodilator were not available. This repeated assessment is a major strength of our study, which enabled us to study TAC in relation to change in lung function. Additionally, IOS, a method measuring respiratory mechanics in contrast to airway calibre measured by spirometry, has not been described in relation to TAC.

Misclassification of exposure may be present, since TAC values were not available for all food items. Despite this, a FFQ similar to the one used in our study was found to have reasonable validity in adults [38]. The FFQ enquired on usual diet the past 12 months and some misclassification due to difficulty to recall past diet cannot be ruled out entirely. However, information on diet was reported before the assessment of the outcome and misclassification of exposure is likely to be non-differential.

In conclusion, results from this longitudinal study indicate that antioxidant intake may be associated with lung function development among children with asthma. The antioxidant intake in the highest TAC tertile in this study corresponds to current recommendations for the general population to consume five servings of fruits and vegetables per day [39]. Together with previous studies [40], our findings emphasise the importance of dietary recommendations for asthma patients. Given the high prevalence of asthma among children and adolescents, our findings may have important public health implications.

## Supplementary material

10.1183/13993003.00990-2019.Supp1 **Please note:** supplementary material is not edited by the Editorial Office, and is uploaded as it has been supplied by the author.
Supplementary material ERJ-00990-2019.SUPPLEMENT

## Shareable PDF

10.1183/13993003.00990-2019.Shareable1 This one-page PDF can be shared freely online.
Shareable PDF ERJ-00990-2019.Shareable
