# Supplementary material for: Dietary antioxidant intake in school age and lung function development up to adolescence
Source: Eur Respir J. 2020 Feb 20;55(2):1900990. doi: 10.1183/13993003.00990-2019 (PMC7031707; doi:10.1183/13993003.00990-2019)
Supplement: Supplementary file 1 [file ERJ-00990-2019.SUPPLEMENT.pdf]

Supplementary Material

Dietary antioxidant intake in school age and lung function development up to adolescence

Emmanouela Sdona, MD, PhD[1], Jenny Hallberg, PhD[1,2,3], Niklas Andersson[1], Sandra Ekström, PhD[1,4], Susanne Rautiainen, PhD[5,6], Niclas Håkansson, PhD[1], Alicja Wolk, PhD[1,7], Inger Kull, PhD[1,2,3], Erik Melén, MD, PhD[1,2,3] and Anna Bergström, PhD[1,4]

Affiliations: [1] Institute of Environmental Medicine, Karolinska Institutet, Stockholm, Sweden, [2] Sachs' Children's Hospital, Södersjukhuset, Stockholm, Sweden, [3] Department of Clinical Science and Education, Södersjukhuset, Karolinska Institutet, Stockholm, Sweden, [4] Centre for Occupational and Environmental Medicine, Region Stockholm, Stockholm, Sweden, [5] Global and Sexual Health, Department of Public Health Sciences, Karolinska Institutet, Stockholm, Sweden, [6] Division of Preventive Medicine, Brigham and Women's Hospital, Boston, USA, [7] Department of Surgical Sciences, Uppsala University, Uppsala, Sweden

Methods

Lung function measurements

Lung function was measured by spirometry at 8 years (n=1832) using a 2200 Pulmonary Function Laboratory (SensorMedics, Anaheim, California, USA) and by impulse oscillometry (IOS) (n=2452) followed by spirometry at 16 years (n=2056) using a Jaeger MasterScreen-IOS system (Carefusion Technologies, San Diego, California, USA). The same spirometry test protocol was used at both time-points, including calibration and quality criteria. All participants performed at least three maximal expiratory flow volume (MEFV) recordings in the sitting position, wearing a nose clip. The highest values of forced expiratory volume in one second (FEV1) and forced vital capacity (FVC) were extracted and used for analysis, provided that the subject's effort was accepted as being maximal by the test leader, the MEFV curve passed visual quality inspection and the two highest FEV1 and FVC readings were reproducible according to American Thoracic Society/ European Respiratory Society (ATS/ERS) criteria (1).

IOS is a noninvasive effort-independent method, which measures respiratory system impedance by superimposing external pressure impulses generated from a loudspeaker, producing recordable waveforms. Measurement was performed during tidal breathing with the lips tightly sealed around the

mouthpiece and supporting cheeks with their hands. Signals free from artefacts that lasted for at least 20 seconds were saved for analysis. At least two recordings were performed per subject. Quality control was performed at the time for examination by visual inspection of the waveforms, and given that coherence, which is a measure of testing reliability, was >0.80 at 10 Hertz (Hz), the mean value of resistance at 5 and 20 Hz (R_5, R_{20}), frequency dependence of resistance (R_{5-20}) and the square root of the area of reactance ($AX^{0.5}$) were used for analysis. The IOS software extracts resistance (R) and reactance (X) in the range of 3-35 Hz from the impedance. Resistance at a low frequency (e.g. R_5) is thought to represent properties of both proximal and distal airways, while resistance at higher frequency (e.g. R_{20}) represents proximal airway resistance. Frequency dependence of resistance i.e. R_{5-20}, may thus represent distal (small) airway resistance (2). The IOS test was performed before spirometry according to clinical standards. The IOS system was calibrated each day using a 3l precision syringe and a reference resistance (0.20 kPa/l/s).

Fractional exhaled nitric oxide (FE_{NO}, expressed as parts per billion, ppb) is a non-invasive test and a biomarker denoting eosinophilic (Th2 driven) lower airway inflammation. Measurements of FE_{NO} (n=2087) were performed at 16 years at an expiratory flow of 50 mL/s ($FE_{NO}50$), using an online chemiluminescent (CLD88) analyser (Eco Medics AG, Duernten, Switzerland), according to ERS/ATS guidelines (3).

Asthma definitions

Asthma was defined based on the parental questionnaire at age 8 years as more than 3 episodes of wheeze in the last 12 months AND/OR at least 1 episode of wheeze in the last 12 months, in combination with inhaled steroids occasionally or regularly. *Inhalant IgE sensitisation* was defined as positive phadiatop test (IgE-value ≥ 0.35 kU/l). *Food IgE sensitisation* was defined as positive fx5 test (IgE-value ≥ 0.35 kU/l).

<u>Sensitivity analyses</u> (Table S7): *Any wheeze* was defined as at least 1 episode of wheeze in the last 12 months. *Doctor's diagnosis of asthma* was defined as doctor's diagnosis of asthma ever in life up to the date of questionnaire 8 or 16. Current asthma was defined as doctor's diagnosis of asthma ever combined with wheeze in the last 12 months at 8 or 16 years. Finally, *according to GALEN/MEDALL* (4), *asthma* was defined as at least TWO of the following three criteria at 8 years:

1. Symptoms of wheeze in the last 12 months prior to the date of questionnaire 8
2. Ever doctor's diagnosis of asthma
3. Asthma medicine occasionally or regularly last 12 months

Other definitions

Parental allergic disease: Mother and/or father with physician-diagnosed asthma and asthma medication and/or physician-diagnosed hay fever in combination with furred pets allergy and/or pollen allergy at the time of baseline questionnaire.

Maternal smoking during pregnancy: The mother smoked at least one cigarette per day in any point of time during the pregnancy.

Parental smoking during infancy: Any of the parents smoked at least one cigarette/day at the time of questionnaire 0.

Socioeconomic status: Socioeconomic status at birth for the household according to dominance order in 2 classes (low: blue collar vs. high: white collar worker).

Education: Education level of the household at baseline in 3 levels (1: elementary school, 2: high school, 3: university).

Statistical analyses

IOS results were not transformed to z-scores, due to lack of reference values, but adjusted for height and age. IOS and FE_{NO} were analyzed on the median using quantile regression, due to non-normally distributed data.

Mixed-effect models for longitudinal data take the correlation between repeated measurements on the same individual into account.

Children with missing data on one of the exposures, outcomes or covariates were not included in that specific analysis, except for the mixed-effect models analyses which require data on the outcome from at least one time-point (see exact number in each table).

Results

Figure S1. Flow chart of the inclusion to the study population. FFQ: food frequency questionnaire, SD: standard deviation

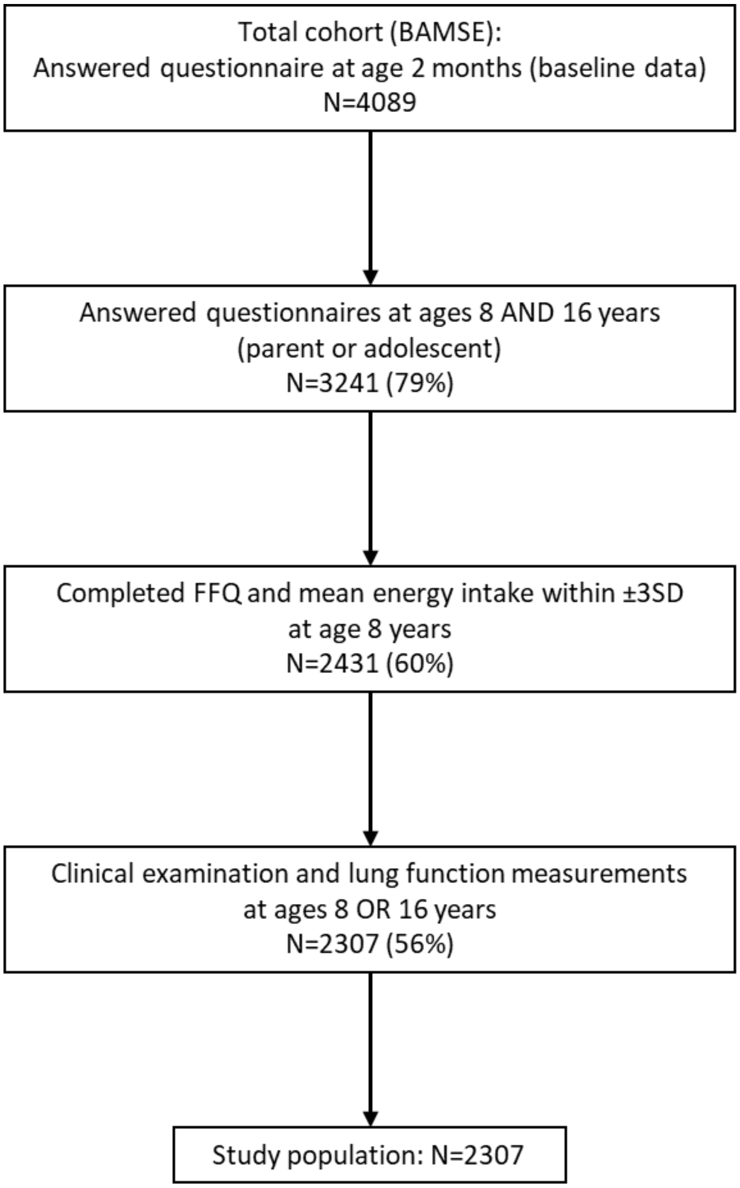

Table S1. Distribution of selected characteristics among children included and excluded from the study population

Selected variables	Children included in the study population N=2307		Children excluded from the study population N=1782		
▪ ***Categorical variables***	***n***	***%***	***n***	***%***	***p-value***[2]
**Boys**	1151	49.9	914	51.3	0.375
**Parental allergic disease**	726	31.7	474	27.0	0.001
**Maternal smoking during pregnancy**	271	11.8	256	14.4	0.013
**Parental smoking during infancy**	471	20.5	384	21.7	0.356
**High socioeconomic status**	1956	85.8	1367	78.7	<0.001
**University education**	1273	55.2	888	50.0	<0.001
**Either parent born out of Sweden**	485	21.1	233	21.1	0.989
**Caesarean section**[1]	271	12.0	236	13.5	0.153
**Exclusive breastfeeding ≥4 months**	1846	81.4	1270	76.9	<0.001
**Older siblings**	1080	46.8	900	50.5	0.018
▪ ***Continuous variables***	***Mean***	***SD***	***Mean***	***SD***	***p-value***[3]
**Maternal age (y)**	31.0	4.5	30.3	4.5	<0.001
**Birth weight**[1] **(g)**	3538	548	3531	557	0.695
**Gestational age**[1] **(w)**	39.5	1.8	39.5	1.8	0.625

95% CI: 95% confidence interval, SD: standard deviation. Numbers may not add up to total due to missing.

[1] Variables obtained from the medical birth register, [2] p-values obtained from the chi-square test, [3] p-values obtained from the *t*-test.

Table S2. Distribution of anthropometric and lung function characteristics among children in the 8-year (n=2307) and 16-year (n=2094) examination

	8 years						16 years					
	Girls			Boys			Girls			Boys		
	**n**	**mean**	**SD**	**n**	**mean**	**SD**	**n**	**mean**	**SD**	**n**	**mean**	**SD**
						**Anthropometric characteristics**						
**Age, y**	1156	8.3	0.5	1151	8.4	0.5	1056	16.7	0.4	1038	16.7	0.4
**Height, m**	1156	1.32	0.06	1151	1.33	0.06	1054	1.67	0.06	1038	1.80	0.07
**Weight, kg**	1156	30.0	5.5	1151	30.4	5.3	1056	60.8	9.1	1038	70.4	11.7
						**Lung function characteristics**						
**FEV_1, ml**	869	1735.2	256.9	839	1824.6	279.5	900	3484.3	435.4	775	4494.5	652.9
**FEV_1, z-score**	868	0.48	0.95	839	0.37	0.92	898	-0.03	0.91	775	-0.03	0.98
**FVC, ml**	869	1990.9	294.8	839	2149.0	341.0	900	4040.2	513.7	775	5389.6	775.9
**FVC, z-score**	869	0.63	0.91	839	0.57	0.91	898	0.17	0.89	775	0.17	0.96
**FEV_1/FVC, %**	869	87.3	5.3	839	85.2	5.9	900	86.5	6.1	775	83.7	6.7
**FEV_1/FVC, z-score**	869	-0.34	0.89	839	-0.35	0.90	898	-0.37	0.94	775	-0.32	0.98
	**n**	**median**	**range**	**n**	**median**	**range**	**n**	**median**	**IQR**	**n**	**median**	**IQR**
**R_5, $Pa{\cdot}L^{-1}{\cdot}s$**							1004	395	105	993	320	90
**R_{20}, $Pa{\cdot}L^{-1}{\cdot}s$**							1004	375	90	993	305	75
**$R_{5\text{-}20}$, $Pa{\cdot}L^{-1}{\cdot}s$**							1004	20.0	55.0	993	15.0	50.0
**$AX^{0.5}$, $(Pa{\cdot}L^{-1})^{0.5}$**							1004	16.4	5.8	992	12.7	5.2
**FE_{NO}, ppb**							836	14.1	10.5	842	17.6	13.8
**Blood eosinophils (10^9 cells/L)**							984	0.1	0.1	979	0.2	0.1
**Blood neutrophils (10^9 cells/L)**							984	3.5	1.6	979	3.0	1.4

SD: standard deviation, IQR: interquartile range, FEV1: forced expiratory volume in 1 sec, FVC: forced vital capacity, R5: mean value of resistance at 5 Hz, R20: mean value of resistance at 20 Hz, R5-20: mean value of resistance between 5 Hz and 20 Hz, AX0.5: square root of the area under the reactance curve, FE_{NO}: fractional exhaled nitric oxide at an expiratory flow of 50 mL/s

Table S3. Distribution of lung function characteristics in girls (n=1156) and boys (n=1151) by tertiles of the TAC of the diet (T1, T2, T3)

	Girls			Boys		
	Tertiles of the TAC of the diet					
	T1	T2	T3	T1	T2	T3
	n=386	n=385	n=385	n=384	n=384	n=383
	**Spirometry at 8 years**					
	n=300	n=292	n=277	n=290	n=280	n=269
	mean (SD)	mean (SD)	mean (SD)	mean (SD)	mean (SD)	mean (SD)
**FEV_1, ml**	1728 (242)	1732 (258)	1746 (271)	1820 (278)	1836 (290)	1818 (270)
**FEV_1, z-score**	0.45 (0.89)	0.46 (0.98)	0.52 (0.98)	0.28 (0.94)	0.41 (0.94)	0.43 (0.87)
**FVC, ml**	1989 (269)	1985 (296)	2000 (320)	2149 (344)	2164 (355)	2133 (323)
**FVC, z-score**	0.64 (0.85)	0.61 (0.94)	0.65 (0.93)	0.49 (0.93)	0.62 (0.94)	0.60 (0.86)
**FEV_1/FVC, %**	87.0 (5.4)	87.4 (5.2)	87.6 (5.5)	85.0 (6.1)	85.1 (5.9)	85.5 (5.7)
**FEV_1/FVC, z-score**	-0.39 (0.89)	-0.34 (0.85)	-0.30 (0.92)	-0.37 (0.93)	-0.36 (0.90)	-0.32 (0.88)
	**Spirometry at 16 years**					
	n=291	n=301	n=308	n=257	n=256	n=262
	mean (SD)	mean (SD)	mean (SD)	mean (SD)	mean (SD)	mean (SD)
**FEV_1, ml**	3495 (468)	3479 (421)	3480 (418)	4503 (709)	4523 (616)	4458 (630)
**FEV_1, z-score**	-0.04 (0.96)	-0.04 (0.87)	-0.02 (0.90)	-0.10 (1.04)	-0.01 (0.95)	0.02 (0.93)
**FVC, ml**	4068 (533)	4017 (511)	4036 (499)	5416 (815)	5433 (762)	5321 (747)
**FVC, z-score**	0.19 (0.91)	0.13 (0.86)	0.18 (0.91)	0.12 (0.99)	0.20 (0.96)	0.20 (0.94)
**FEV_1/FVC, %**	86.1 (6.3)	86.8 (5.8)	86.5 (6.3)	83.4 (7.0)	83.6 (6.8)	84.0 (6.2)
**FEV_1/FVC, z-score**	-0.42 (0.95)	-0.32 (0.90)	-0.36 (0.98)	-0.35 (1.02)	-0.32 (0.99)	-0.29 (0.92)
	**IOS at 16 years**					
	n=324	n=339	n=341	n=331	n=328	n=334
	median (IQR)	median (IQR)	median (IQR)	median (IQR)	median (IQR)	median (IQR)
**R_5, $Pa \cdot L^{-1} \cdot s$**	395 (100)	400 (110)	395 (105)	325 (95)	322.5 (90)	320 (90)
**R_{20}, $Pa \cdot L^{-1} \cdot s$**	375 (85)	370 (100)	375 (80)	310 (75)	305 (75)	305 (70)
**R_{5-20}, $Pa \cdot L^{-1} \cdot s$**	20.0 (52.5)	20.0 (60.0)	20.0 (55.0)	15.0 (50.0)	15.0 (50.0)	15.0 (40.0)
**$AX^{0.5}$, $(Pa \cdot L^{-1})^{0.5}$**	16.1 (5.8)	16.7 (6.0)	16.6 (6.0)	12.6 (5.4)	12.5 (5.0)	12.6 (4.8)
	**Additional parameters at 16 years**					

	**n=273**	**n=283**	**n=280**	**n=273**	**n=282**	**n=287**
**FE_{NO} (ppb)**	13.6 (10.6)	13.8 (11.9)	14.8 (9.5)	17.8 (15.2)	17.6 (14.7)	17.3 (12.9)
	**n=321**	**n=332**	**n=331**	**n=320**	**n=328**	**n=331**
**Blood eosinophils (10^9 cells/L)**	0.1 (0.1)	0.1 (0.1)	0.1 (0.1)	0.2 (0.1)	0.2 (0.1)	0.2 (0.1)
**Blood neutrophils (10^9 cells/L)**	3.5 (1.6)	3.6 (1.6)	3.5 (1.6)	3.0 (1.3)	2.9 (1.5)	3.0 (1.4)

Table S4. Associations between TAC in tertiles (T1 reference, T2, T3) at 8 years and spirometry results at 8 and 16 years by sex

	8 years				16 years			
	Girls (n=869)		Boys (n=839)		Girls (n=900)		Boys (n=775)	
	β	95% CI	β	95% CI	β	95% CI	β	95% CI
**FEV1, ml**								
T1	reference		reference		reference		reference	
T2	5.9	-24.3-36.0	25.5	-6.1-57.1	-11.0	-71.7-49.7	42.5	-49.5-134.4
T3	14.3	-16.6-45.3	31.6	-0.1-63.4	-5.1	-65.8-55.7	60.7	-31.3-152.7
p for trend		0.477		0.131		0.888		0.231
**FVC, ml**								
T1	reference		reference		reference		reference	
T2	-1.8	-35.7-32.1	24.8	-12.5-62.1	-35.9	-105.9-34.0	37.4	-66.3-141.2
T3	7.3	-27.5-42.2	28.3	-9.2-65.8	-13.9	-83.9-56.1	50.6	-53.3-154.4
p for trend		0.601		0.172		0.918		0.296
**FEV1/FVC, %**								
T1	reference		reference		reference		reference	
T2	0.4	-0.5-1.3	0.2	-0.7-1.2	0.5	-0.5-1.5	0.3	-0.9-1.5
T3	0.5	-0.4-1.4	0.3	-0.7-1.3	0.2	-0.8-1.2	0.3	-0.8-1.5
p for trend		0.534		0.867		0.849		0.712

Spirometry data were analyzed by linear regression on the mean, adjusted for height and age at examination, maternal smoking during pregnancy, parental smoking during infancy, parental allergic disease, socioeconomic status, older siblings and maternal age < 26 years.

Table S5. Associations between TAC (tertiles 2 and 3 combined vs reference tertile 1) at 8 years and adjusted spirometry results at 8 and 16 years stratified by asthma at 8 years

		FEV1, ml		FVC, ml		FEV1/FVC, %	
	n	β	95% CI	β	95% CI	β	95% CI
**8 years**							
**No asthma**	1526	8.3	-11.7-28.3	7.4	-15.6-30.3	0.1	-0.5-0.7
**Asthma**	134	31.4	-41.9-104.8	33.1	-57.6-123.7	0.4	-1.9-2.6
p for interaction		0.525		0.524		0.870	
**16 years**							
**No asthma**	1501	-7.3	-57.2-42.6	-6.4	-62.9-50.2	0.1	-0.6-0.8
**Asthma**	124	200.0	38.3-361.6	85.9	-126.2-297.9	2.5	-0.2-5.1
p for interaction		0.018		0.353		0.070	

Linear regression on the mean adjusted for sex, height and age at examination, maternal smoking during pregnancy, parental smoking during infancy, parental allergic disease, socioeconomic status, older siblings and maternal age < 26 years.

Table S6. Associations between TAC (tertiles 2 and 3 combined vs reference tertile 1) at 8 years and adjusted spirometry results at 8 and 16 years for the total population and excluding supplement users, stratified by asthma at 8 years

		**FEV1, z-score**		**FVC, z-score**		**FEV1/FVC, %**	
	**n**	**β**	**95% CI**	**β**	**95% CI**	**β**	**95% CI**
**Total population**							
**No asthma**	1948						
8 years		0.03	-0.07-0.12	0.03	-0.07-0.12	0.1	-0.6-0.7
16 years		-0.01	-0.11-0.08	-0.01	-0.11-0.08	0.1	-0.5-0.7
Change		-0.04	-0.14-0.05	-0.04	-0.13-0.05	0.1	-0.5-0.6
**Asthma**	154						
8 years		0.21	-0.12-0.54	0.11	-0.25-0.47	0.8	-1.5-3.0
16 years		0.46	0.11-0.80	0.30	-0.07-0.67	2.2	-0.2-4.6
Change		0.25	-0.07-0.57	0.18	-0.11-0.48	1.4	-1.1-3.9
**Excluding supplement users**							
**No asthma**	1145						
8 years		0.11	-0.01-0.24	0.08	-0.04-0.20	0.3	-0.5-1.1
16 years		0.05	-0.08-0.18	0.04	-0.09-0.16	0.2	-0.6-1.1
Change		-0.06	-0.19-0.06	-0.05	-0.17-0.07	-0.1	-0.8-0.7
**Asthma**	85						
8 years		0.35	-0.10-0.81	0.08	-0.42-0.59	2.6	-0.6-5.7
16 years		0.42	-0.02-0.86	0.07	-0.43-0.56	3.9	0.9-6.9
Change		0.07	-0.38-0.52	-0.02	-0.46-0.42	1.4	-2.1-4.8

β-coefficients and 95% confidence intervals (CI) were estimated using mixed effect models, adjusted for sex, maternal smoking during pregnancy, parental smoking during infancy, parental allergic disease, socioeconomic status, older siblings and maternal age < 26 years.

Table S7. Associations between TAC (tertiles 2 and 3 combined vs reference tertile 1) at 8 years and FEV_1 at 8 and 16 years among children with asthma or wheeze at 8 years

**FEV_1, z-score**	**MeDALL asthma**		**Doctor's diagnosis of asthma up to 8 years**		**Current asthma**		**Any wheeze**	
	**Diff.**	**95% CI**	**Diff.**	**95% CI**	**Diff.**	**95% CI**	**Diff.**	**95% CI**
	**n=232**		**n=149**		**n=138**		**n=241**	
**8 years**	0.07	-0.18-0.33	-0.05	-0.36-0.25	0.16	-0.16-0.48	0.13	-0.13-0.40
**16 years**	0.27	0.00-0.54	0.30	-0.02-0.61	0.50	0.16-0.84	0.33	0.06-0.60
**Overall**	0.16	-0.06-0.39	0.11	-0.16-0.37	0.31	0.03-0.59	0.22	-0.01-0.46
**Change**	0.20	-0.07-0.46	0.35	0.03-0.67	0.34	0.00-0.68	0.20	-0.06-0.46

β-coefficients and 95% confidence intervals (CI) were estimated using mixed effect models, adjusted for sex, maternal smoking during pregnancy, parental smoking during infancy, parental allergic disease, socioeconomic status, older siblings and maternal age < 26 years.

Interaction terms: MeDALL asthma p=0.284, time-specific p=0.088, Doctor's diagnosis of asthma up to 8 years p=0.737, time-specific p=0.020, Current asthma p=0.086, time-specific p=0.024, Any wheeze p=0.153, time-specific p=0.068

Table S8. Associations between TAC (in tertiles, T1 reference) at 8 years and IOS and FE_{NO} results at 16 years

	T1	T2				T3			
		Girls		Boys		Girls		Boys	
	β	β	95% CI	β	95% CI	β	95% CI	β	95% CI
IOS results (n=1004 girls and n=993 boys)									
**R_5, Pa·L-^{1}·s**	0	0.5	-13.9-14.9	-6.7	-19.1-5.7	-10.3	-24.8-4.3	-10.8	-23.2-1.7
**R_{20}, Pa·L^{-1}·s**	0	-5.0	-18.4-8.4	-0.4	-10.1-9.4	-6.9	-20.4-6.7	-7.4	-17.2-2.3
**$R_{5\text{-}20}$, Pa·L^{-1}·s**	0	-3.0	-10.9-5.0	1.2	-5.8-8.1	-5.7	-13.8-2.3	-4.5	-11.4-2.5
**$AX^{0.5}$, (Pa·L^{-1})$^{0.5}$**	0	0.3	-0.5-1.1	0.2	-0.5-0.8	0.1	-0.7-1.0	0.1	-0.6-0.7
Additional parameters (n=836 girls and n=842 boys)									
**FE_{NO}, ppb**	0	0.02	-1.5-1.5	-0.3	-2.4-1.7	1.2	-0.4-2.7	0.4	-1.6-2.5

IOS and FE_{NO} data were analyzed by linear regression on the median, adjusted for height and age at examination, maternal smoking during pregnancy, parental smoking during infancy, parental allergic disease, socioeconomic status, older siblings and maternal age < 26 years.

References

1. Miller M, Hankinson J, Brusasco V, Burgos F, Casaburi R, Coates A, et al. Standardisation of spirometry. Eur Respir J. 2005;26(2):319-38.
2. Goldman M, Saadeh C, Ross D. Clinical applications of forced oscillation to assess peripheral airway function. Respir Physiol Neurobiol. 2005;148(179-194).
3. American Thoracic Society/European Respiratory Society. ATS/ERS recommendations for standardized procedures for the online and offline measurement of exhaled lower respiratory nitric oxide and nasal nitric oxide, 2005. Am J Respir Crit Care Med. 2005;171(8):912-30.
4. Pinart M, Benet M, Annesi-Maesano I, von Berg A, Berdel D, Carlsen K, et al. Comorbidity of eczema, rhinitis, and asthma in IgE-sensitised and non-IgE-sensitised children in MeDALL: a population-based cohort study. Lancet Respir Med. 2014;2(2):131-40.
