# Supplementary material for: Dietary antioxidant intake in school age and lung function development up to adolescence
Source: Eur Respir J. 2020 Feb 20;55(2):1900990. doi: 10.1183/13993003.00990-2019 (PMC7031707; doi:10.1183/13993003.00990-2019)
Supplement: Supplementary file 2 [file ERJ-00990-2019.Shareable.pdf]

SHAREABLE PDF

# Dietary antioxidant intake in school age and lung function development up to adolescence

Emmanouela Sdona [1], Jenny Hallberg [1,2,3], Niklas Andersson [1], Sandra Ekström [1,4], Susanne Rautiainen [5,6], Niclas Håkansson [1], Alicja Wolk [1,7], Inger Kull [1,2,3], Erik Melén [1,2,3] and Anna Bergström [1,4]

**Affiliations**: [1]Institute of Environmental Medicine, Karolinska Institutet, Stockholm, Sweden. [2]Sachs' Children's Hospital, Södersjukhuset, Stockholm, Sweden. [3]Dept of Clinical Science and Education, Södersjukhuset, Karolinska Institutet, Stockholm, Sweden. [4]Centre for Occupational and Environmental Medicine, Region Stockholm, Stockholm, Sweden. [5]Global and Sexual Health, Dept of Public Health Sciences, Karolinska Institutet, Stockholm, Sweden. [6]Division of Preventive Medicine, Brigham and Women's Hospital, Boston, MA, USA. [7]Dept of Surgical Sciences, Uppsala University, Uppsala, Sweden.

**Correspondence**: Emmanouela Sdona, Institute of Environmental Medicine, Karolinska Institutet, Box 210, SE-171 77 Stockholm, Sweden. E-mail: emmanouela.sdona@ki.se

@ERSpublications
**Dietary antioxidant intake at school age may influence lung function development as measured by $FEV_1$ up to adolescence among children with asthma. In contrast, no association was observed among children without asthma.** http://bit.ly/2CzEZ8W

**Cite this article as:** Sdona E, Hallberg J, Andersson N, *et al.* Dietary antioxidant intake in school age and lung function development up to adolescence. *Eur Respir J* 2020; 55: 1900990 [https://doi.org/10.1183/13993003.00990-2019].

This single-page version can be shared freely online.

ABSTRACT Dietary antioxidant intake has been hypothesised to influence lung function. The association between total antioxidant capacity (TAC) of the diet at age 8 years and lung function development up to 16 years in 2307 participants from the Swedish population-based birth cohort BAMSE (Children, Allergy, Milieu, Stockholm, Epidemiology) was investigated.

Information on TAC was obtained from a food frequency questionnaire at 8 years. Lung function was measured by spirometry at 8 and 16 years, impulse oscillometry (IOS) and exhaled nitric oxide fraction ($F_{eNO}$) at 16 years. Low lung function was defined as forced expiratory volume in 1 s ($FEV_1$) z-score below the 25th percentile. Longitudinal associations between TAC and lung function were analysed by mixed effect models adjusted for potential confounders. Stratification by asthma at 8 years was performed to examine effect modification.

The median TAC intake was 10 067 μmol Trolox equivalents (TE)·$g^{-1}$, with males having a lower mean compared to females (9963 *versus* 10 819 μmol TE·$g^{-1}$). In analyses of lung function change between 8 and 16 years, there were no statistically significant associations between TAC in tertiles and spirometry results for the total study population. Among children with asthma at 8 years (prevalence 7%), higher TAC was associated with higher mean $FEV_1$ (0.46 SD, 95% CI 0.11–0.80) and decreased odds of low lung function at 16 years (OR 0.28, 95% CI 0.12–0.65). There were no associations between TAC and forced vital capacity or IOS/$F_{eNO}$ results.

High dietary antioxidant intake in school age may be associated with improved lung function development from school age to adolescence among children with asthma.

Copyright ©ERS 2020. This article is open access and distributed under the terms of the Creative Commons Attribution Licence 4.0.
